# Impaired Duration Perception in Patients With Unilateral Vestibulopathy During Whole-Body Rotation

**DOI:** 10.3389/fnint.2022.818775

**Published:** 2022-06-03

**Authors:** Eunjin Kwon, Ju-Young Lee, Jung-Mi Song, Hyo-Jung Kim, Jong-Hee Lee, Jeong-Yoon Choi, Ji-Soo Kim

**Affiliations:** ^1^Department of Neurology, Chungnam National University Hospital, Daejeon, South Korea; ^2^Department of Neurology, Kangdong Sacred Heart Hospital, Hallym University College of Medicine, Seoul, South Korea; ^3^Research Administration Team, Seoul National University Bundang Hospital, Seongnam-si, South Korea; ^4^Dizziness Center, Seoul National University Bundang Hospital, Seongnam-si, South Korea; ^5^Department of Neurology, Clinical Neuroscience Center, Seoul National University Bundang Hospital, Seoul, South Korea; ^6^Department of Neurology, Seoul National University College of Medicine, Seongnam-si, South Korea

**Keywords:** vestibular perception, unilateral vestibulopathy, whole-body rotation, duration perception, spatial navigation

## Abstract

This study aimed to evaluate vestibular perception in patients with unilateral vestibulopathy. We recruited 14 patients (9 women, mean age = 59.3 ± 14.3) with unilateral vestibulopathy during the subacute or chronic stage (disease duration = 6 days to 25 years). For the evaluation of position perception, the patients had to estimate the position after whole-body rotation in the yaw plane. The velocity/acceleration perception was evaluated by acquiring decisions of patients regarding which direction would be the faster rotation after a pair of ipsi- and contra-lesional rotations at various velocity/acceleration settings. The duration perception was assessed by collecting decisions of patients for longer rotation directions at each pair of ipsi- and contra-lesional rotations with various velocities and amplitudes. Patients with unilateral vestibulopathy showed position estimates and velocity/acceleration discriminations comparable to healthy controls. However, in duration discrimination, patients had a contralesional bias such that they had a longer perception period for the healthy side during the equal duration and same amplitude rotations. For the complex duration task, where a longer duration was assigned to a smaller rotation amplitude, the precision was significantly lower in the patient group than in the control group. These results indicate persistent impairments of duration perception in unilateral vestibulopathy and favor the intrinsic and distributed timing mechanism of the vestibular system. Complex perceptual tasks may be helpful to disclose hidden perceptual disturbances in unilateral vestibular hypofunction.

## Introduction

The vestibular apparatus anchored in the inner ear generates neural signals related to acceleration, velocity, and duration of head motion (Angelaki and Cullen, 2008; Diaz-Artiles and Karmali, 2021). In the brain, the vestibular signals interact with other sensory cues such as vision and proprioception, thereby enabling the motion perception and spatial representation of the head (Seemungal, 2014; Diaz-Artiles and Karmali, 2021). In addition, the vestibular signals generate ocular, spinal, and autonomic reflexes (Angelaki and Cullen, 2008; Kwon et al., 2021). Therefore, with vestibular dysfunction, the motion perception and spatial representation of the head may become disturbed along with the appearance of various clinical signs. Clinically, acute unilateral vestibulopathy is one of the most common vestibular dysfunctions. Patients usually report compelling vertigo (false motion sense) and show nystagmus, postural imbalance, and autonomic disturbances (Bronstein and Dieterich, 2019; Kim, 2020). The symptoms and signs decrease over time, but there may be substantial individual differences in the timing and extent of recovery (Best et al., 2009; Halmagyi et al., 2010). In fact, many patients have persistent dizziness and imbalance in the subacute and chronic stages, symptoms related to the vestibulo-perceptual (VP) pathway, without other objective signs in the vestibulo-ocular and vestibulo-spinal reflex pathways (Staab et al., 2017).

Hence, there have been several attempts to characterize the VP in those patients distinct from healthy individuals. In vestibular threshold tests, healthy individuals had a higher threshold for VP than for VOR (Seemungal et al., 2004). Patients with acute unilateral vestibulopathy had increased VP and VOR thresholds in the acute phase (i.e., they become less sensitive to vestibular stimulation) (Cousins et al., 2013). Both tended to recover over time, but the threshold of VP regained a symmetricity between the rotation toward the lesion side and the healthy side, while that of VOR did not (Cousins et al., 2013). In position estimation tests with rotational vestibular stimuli, healthy individuals tended to underestimate with a gain of about 0.8–0.9 (Kaski et al., 2016; Choi et al., 2021). In a similar experimental setting, patients with unilateral vestibulopathy also had a position estimate comparable to healthy individuals in both acute and chronic stages (Cohen et al., 2017). Regarding duration perception, a decreased duration perception for motion in the acute phase of unilateral vestibulopathy nearly recovered in the chronic phase (Cousins et al., 2013). These results may imply a strong resilience of the VP pathway but may not account for the long-lasting perceptual disturbance of patients with unilateral vestibulopathy, so further studies are warranted.

Patients with vestibulopathy may still have minor perceptual errors, which may have worked together with risk factors, such as visual dependence and psychological disturbances, to cause persistent vertigo (Best et al., 2009; Cousins et al., 2017). In fact, a recent study with a more complex rotational task, e.g., a repetitive asymmetric rotation task, revealed a biased spatial representation of the head (Panichi et al., 2017). On the contrary, the neural noise in VP and VOR pathways is proportional to the stimulus intensity (Nouri and Karmali, 2018). Therefore, the perceptual disturbance may partly be owed to the amplified noisy signals after recovery from illness, and this point of view highlights the need for precision evaluation for vestibular perception. Of interest, the precision of duration perception in the complex task was significantly altered, especially for elderly patients (Choi et al., 2021). However, further studies on patients with unilateral vestibulopathy are necessary to verify the explanation. In these backgrounds, this study investigated the characteristics of VP in patients with unilateral vestibulopathy after recovering from acute illness.

## Materials and Methods

### Standard Protocol Approvals, Registrations, and Patient Consent

The Institutional Review Board of Seoul National University Bundang Hospital approved this prospective experimental study (B-1908-556-301), and written informed consent was taken from all patients before the experiment.

### Patients and Controls

From September 2019 to February 2020, we recruited 14 patients (9 women, mean age = 59.3 ± 14.3) with unilateral vestibulopathy in the subacute and chronic stages (with a symptom duration ranging from 6 to 25 years, median = 25 days). Patients underwent complete neurological and neuro-otological examinations. We defined unilateral vestibulopathy as the patients having a positive unilateral head impulse test (video head impulse gain < 0.7) or unilateral caloric paresis (>20% on bithermal caloric test). For the included patients, the mean ipsilesional head impulse gain was 0.60 ± 0.26, and the mean caloric paresis was 64.6% ± 30.75%. They had no abnormal symptoms or signs indicative of central nervous system disorders. All patients underwent a mini-mental state examination (mean score = 28.9 ± 1.7). The clinical characteristics of the included patients are presented in Table 1. For the comparison, we made a control set comprised of 14 age-matched healthy subjects evaluated with the same experimental protocols (Choi et al., 2021).

**TABLE 1:** Clinical characteristics of included patients.

Patient	Age	Sex	Lesion location	Cause of vestibulopathy	Duration	Head impulse gain		Caloric paresis
						CLHC	ILHC	
1	65	M	Left	Vestibular schwannoma	3 years	1.31	0.58	−68
2	69	F	Left	Vestibular Neuritis	17 days	1.11	0.58	−58
3	73	M	Left	Vestibulopathy	1 year	0.98	0.36	−100
4	82	F	Left	Vestibulopathy	6 days	1.05	0.93	−93
5	48	F	Left	Vestibulopathy	9 days	1.16	0.64	−39
6	72	F	Left	Vestibulopathy	6 days	0.91	0.66	−9
7	59	M	Left	Vestibulopathy	3 months	0.88	0.43	−100
8	65	F	Left	Vestibular schwannoma	5 years	1.06	1.01	−49
9	48	M	Right	Vestibular Neuritis	1 month	0.71	0.31	N/A
10	40	F	Left	Vestibulopathy	6 months	1.18	1.14	−41
11	69	F	Left	Vestibulopathy	14 days	0.96	0.29	−89
12	30	F	Left	Vestibular Neuritis	7 days	0.89	0.44	N/A
13	55	F	Left	CPA tumor	15 days	0.85	0.65	N/A
14	55	M	Left	Vestibulopathy	25 years	0.90	0.40	N/A

*N/A, data not available; CPA, cerebellopontine angle; CLHC, contralesional horizontal canal; ILHC, ipsilesional horizontal canal. A negative value in caloric paresis indicates left side caloric paresis, whereas a positive one refers to right side caloric paresis.*

### Experimental Apparatus

For the experiments, we adopted the motorized chair, rotating at various constant velocities with 0.15 s of fixed acceleration and deceleration periods. According to the targeted velocity, the acceleration and deceleration ranged from 100°/s^2^ to 800°/s^2^. The amplitude and duration of rotation were predetermined according to the experimental paradigms, and the experimenter entirely controlled the chair.

### Tasks for Vestibular Perception

All patients sat in a chair with a safety belt fastened and underwent rotational experiments while wearing covered goggles and headphones with white noise to prevent visual and auditory cues. After receiving guidance on the method and purpose of each task, patients underwent the experiments in the order of position, velocity/acceleration, and duration tasks.

**FIGURE 1:**
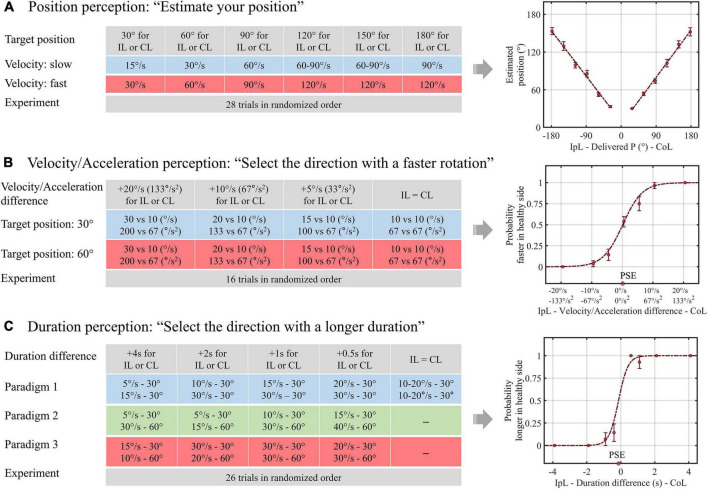
Schematics of the experimental design and data analysis for vestibular perception. **(A)** In paradigm 1 of the duration task, the duration difference was created by delivering equal amplitude rotations with different velocities. In paradigms 2 **(B)** and 3 **(C)**, longer and shorter rotation durations were assigned for larger rotational amplitudes, respectively. IpL, ipsilesional; CoL, contralesional; P, position; PSE, point of subjective equality.

In the position task, we collected a positional estimate of the patients after whole-body rotation. The design of the position task was as follows: the amplitude of whole-body rotation was between 30° and 180° in 30° steps to the right or left, and each rotational position was delivered at two or three velocities ranging from 15 to 120°/s (Figure 1A). After each rotation, patients reported their estimated rotational position and then passively returned to the initial position. There was a 30-s pause between each rotation to prevent the effect of post-rotational cues. All patients were given a practice trial for six rotational positions, with rotations ranging from 30° to 180° in 30° steps. For each practice rotation, patients had auditory feedback on their estimates. Then, each patient underwent 28 rotations without feedback in random order during the position task.

In the velocity/acceleration task, we acquired patients’ choices about the direction of the faster rotation after a pair of left-right rotations. The patients were rotated either rightward or leftward, returned to the initial position, and then rotated in the opposite direction. The rotation velocity was 10, 15, 20, or 30°/s for one direction and 10°/s for the other direction, thereby creating velocity differences of 0, 5, 10, and 20°/s. After each pairwise rotation, the participants reported the “faster” direction. In our experiment, the target velocity reached 0.15 s, so the acceleration was proportional to the velocity. We named it the velocity/acceleration task because it was unclear whether the participants used the velocity or acceleration cues to determine the faster direction. We performed this task at two different rotation amplitudes, namely, small (30°) and large (60°). After two practice trials with feedback for correctness, patients underwent 16 experimental trials without feedback (Figure 1B).

In the duration task, the patients reported the direction of the longer rotation after a pair of left-right rotations. The differences in rotation duration were 0, 0.5, 1, 2, and 4 s. In addition, as introduced in the previous study, we adopted three different paradigms to evaluate whether the interaction between the amplitude and duration of motion would change the duration perception. Therefore, in a pair of left-right rotations of paradigm 1, we applied the same amplitude rotation with different velocities to create a difference in duration. In paradigm 2, we adopted different velocities and amplitudes to assign longer rotation durations for smaller rotational amplitudes, e.g., 0.5 s longer for the rightward rotation was designed by applying 30° rightward rotation at a velocity of 15°/s and 60° leftward rotation at a velocity of 40°/s. Finally, in paradigm 3, we assigned a longer rotation duration to the larger rotational amplitude, e.g., 0.5 s longer for rightward rotation of 60° at a velocity of 30°/s and leftward rotation of 30° at a velocity of 20°/s. Patients had six practice trials with auditory feedback for the duration perception and underwent 26 experimental trials without feedback (Figure 1C).

### Data Management and Statistical Analyses

To investigate the perceptual characteristics of the patient group, we merged the patient data with age-matched control data and treated the group (patients vs. control) as a nominal variable. Due to the limited number of rotations per given stimulus, we analyzed the relationship between actual stimuli and the perceptual responses *via* a generalized linear model (GLM) using pooled group data. In the position data analysis, we adopted a GLM with a linear fit. The slope of regression, β, represents the change of the position estimate in response to a change in the actual stimulus. We compared β during ipsi- and contralesional rotations between the patient and control groups.

In contrast, for the velocity/acceleration and duration tasks, we used a GLM with a logit fit. The intercept value of the regression equation represents the probability of selecting “contralesional rotation was faster or longer” in the rotation without a velocity/acceleration or duration difference between ipsi- and contralesional rotations. β is the change in the logarithm of the odds, *ln*⁡(*p*/1−*p*) in response to the velocity/acceleration or duration difference change. Therefore, the ideal intercept and β values indicate the accuracy and precision of the discriminative ability. Specifically, we analyzed the duration task through two statistical models. Model 1 included only the duration difference (actual stimulus) and group (patient vs. control) as variables, whereas Model 2 further included the velocity difference, creating the duration difference. A *p*-value of less than 0.05 was defined as the level of statistical significance. For the multiple comparisons, we set the *p*-value using the Bonferroni correction.

## Results

### Position Task

**FIGURE 2:**
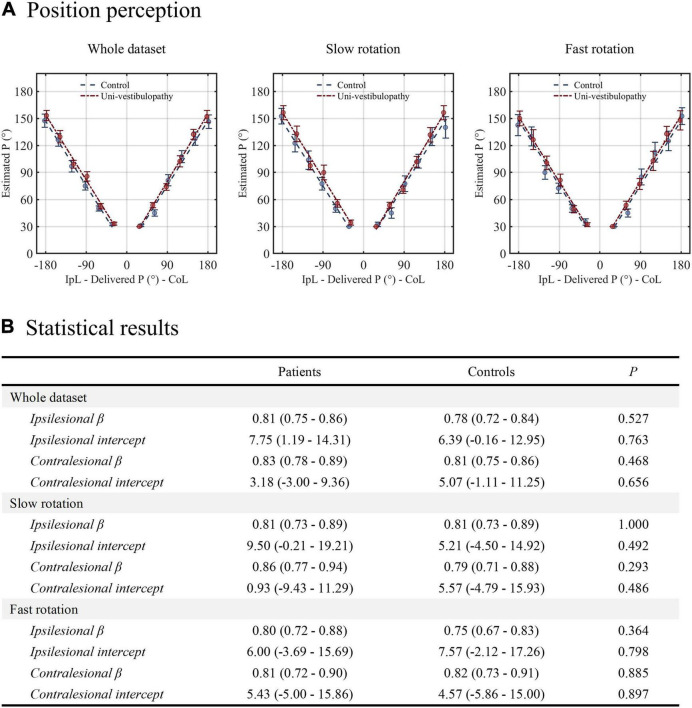
The results of the position task. **(A)** The results from the patient group (red line) are presented above the regression results from the age-matched control group (blue line). **(B)** The results of statistical analyses performed for the position task. Statistical analyses were performed using the generalized linear model with a linear fit. For clarity, the statistical model presented in this figure did not include velocity covariates. β indicates the change in the position estimate in response to the change in the actual stimulus. The intercept value indicates the static positional bias. IpL, ipsilesional; CoL, contralesional.

The results of the position task are presented in Figure 2. For the whole dataset, the GLM with linear fit showed that the regression slope of the patient group was 0.81 for ipsilesional rotation and 0.83 for contralesional rotation. Compared with the control group, the regression slope was not different regardless of the rotational direction (*p*-values for ipsilesional and contralesional rotation were 0.53 and 0.47, respectively). This pattern was reaffirmed in the subgroup analyses according to the adopted velocity (slow vs. fast) for rotation. The regression slopes during the slow or fast rotation paradigm were not different between the patient and control groups, irrespective of the rotational direction (all *p* > 0.05).

### Velocity/Acceleration Task

**FIGURE 3:**
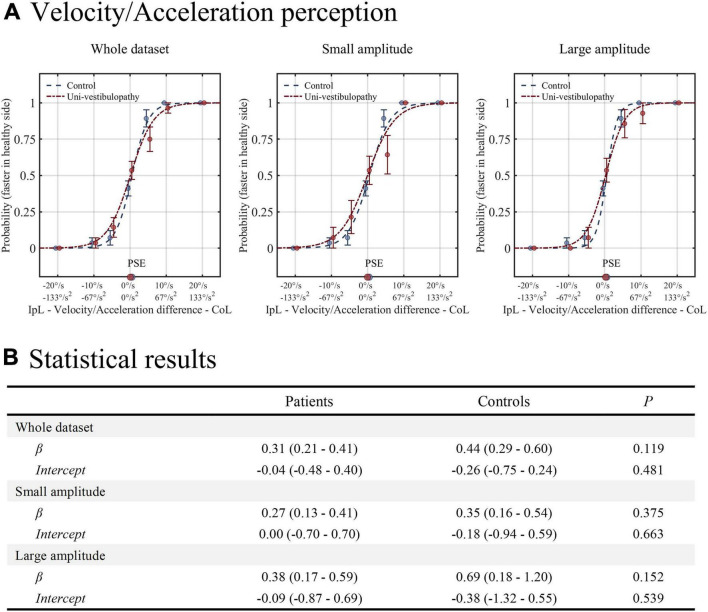
The results of the velocity/acceleration task. **(A)** The results from the patient group (red line) are presented above on the regression results from the age-matched control group (blue line). **(B)** The results of statistical analyses for the velocity/acceleration task. Statistical analyses were performed using the generalized linear model with logit fit. IpL, ipsilesional; CoL, contralesional; PSE, point of subjective equality, which is the stimulus amplitude that corresponds to the 0.5 probability point.

The results of this task are presented in Figure 3. The GLM with a logit fit for the whole dataset showed that the intercept and β values were −0.04 (−0.48–0.40) and 0.31 (0.21–0.41), respectively. Hence, the probability of selecting “contralesional rotation was faster” was 0.49 (0.38–0.60) when equal velocity/acceleration was applied in both directions. The intercept and β values were not different from those of the control group (*p* = 0.48 and 0.12). The findings were similar in small and large amplitudes of rotation (intercept = 0 and –0.09; β = 0.27 and 0.38), which were not different from the control group (all *p* > 0.05).

### Duration Task

**FIGURE 4:**
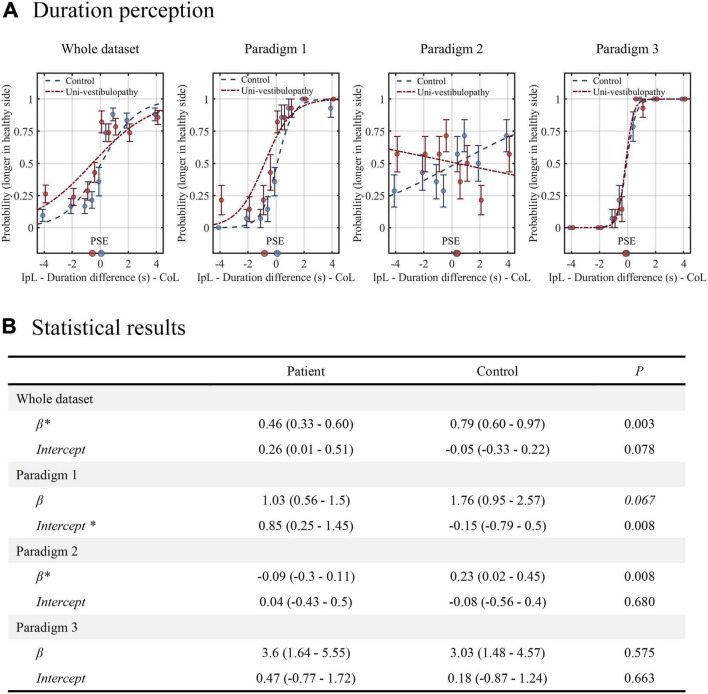
The results of the duration task. **(A)** The results from the patient group (red line) are presented above the regression results from the age-matched control group (blue line). **(B)** The results of statistical analyses for the duration task. Statistical analyses were performed using the generalized linear model with logit fit. For clarity, the statistical model presented in this figure did not include velocity covariates. An asterisk (*) indicates the parameters with statistical significance. The level of statistical significance was set at 0.05 for the whole dataset and 0.0167 for the paradigms 1–3. IpL, ipsilesional; CoL, contralesional; PSE, point of subjective equality, which is the stimulus amplitude that corresponds to the 0.5 probability point.

The results of the duration task are presented in Figure 4 and Table 2. In statistical model 1 for the whole dataset, an intercept was 0.26 (0.01–0.51), and the β value was 0.46 (0.33–0.60). The probability of selecting “contralesional rotation was longer” was estimated to be 0.57 (0.50–0.63) when equal velocity was applied in both directions. Statistically, the intercept tended to be different from that of the control group (*p* = 0.08), while the β value was significantly lower than that of the control group (*p* = 0.003). In addition, the regression results differed significantly by the experimental paradigm.

**TABLE 2:** Statistical analyses for duration perception.

	Model 1		Model 2	
	β with 95% CI	*P*	β with 95% CI	*P*
Whole dataset				
Intercept	−0.05 (−0.32–0.21)	0.691	−0.09 (−0.37–0.20)	0.544
Group (control to patients)	0.31 (−0.05–0.67)	0.079	0.31 (−0.05–0.67)	0.085
ΔDuration	0.79 (0.61–0.97)	<0.001	0.85 (0.64–1.05)	<0.001
ΔVelocity			−0.01 (−0.03–0.00)	0.127
Group×Δduration	−0.32 (−0.54–−0.10)	0.003	−0.34 (−0.56–−0.11)	0.003
ΔDuration×Δvelocity			0.00 (−0.01–0.01)	0.519
Paradigm 1^†^				
Intercept	−0.15 (−0.73–0.44)	0.589	−0.28 (−1.03–0.46)	0.405
Group (control to patients)	0.99 (0.20–1.79)	0.008	1.29 (0.32–2.27)	0.004
ΔDuration	1.76 (1.02–2.49)	<0.001	0.54 (−0.05–1.14)	0.047
ΔVelocity			0.13 (0.07–0.18)	<0.001
Group×Δduration	−0.73 (−1.58–0.12)	0.067	−0.16 (−0.82–0.49)	0.588
ΔDuration×Δvelocity			0.01 (−0.02–0.05)	0.514
Paradigm 2				
Intercept	−0.08 (−0.50–0.35)	0.696	−0.19 (−0.75–0.38)	0.455
Group (control to patients)	0.11 (−0.48–0.71)	0.680	0.11 (−0.50–0.72)	0.697
ΔDuration	0.23 (0.04–0.43)	0.008	0.23 (−0.02–0.47)	0.040
ΔVelocity			0.00 (−0.02–0.02)	0.907
Group×Δduration	−0.33 (−0.59–−0.06)	0.007	−0.33 (−0.60–−0.06)	0.007
ΔDuration×Δvelocity			0.00 (−0.01–0.02)	0.483
Paradigm 3				
Intercept	0.18 (−0.76–1.12)	0.671	0.20 (−0.95–1.35)	0.704
Group (control to patients)	0.29 (−1.16–1.74)	0.663	0.30 (−1.22–1.83)	0.657
ΔDuration	3.03 (1.65–4.40)	<0.001	2.86 (1.40–4.31)	<0.001
ΔVelocity			0.03 (−0.08–0.13)	0.572
Group×Δduration	0.57 (−1.64–2.78)	0.575	0.53 (−1.64–2.69)	0.589
ΔDuration×Δvelocity			−0.00 (−0.20–0.20)	0.983

*We adopted a generalized linear model with a logit fit for the statistical analyses. In model 1, the included variables were the group (control vs. patient) and duration difference (Δ) with an interaction term. In model 2, velocity difference (Δ) was also included as a covariate.*

*^†^Note that statistical model 2 in paradigm 1 showed the Δvelocity had the most significant p-value, indicating the duration perception depends on Δvelocity rather than Δduration. In contrast, Δvelocity was revealed not to affect the duration perception in other paradigms. The level of statistical significance was set at 0.05 for the whole dataset and 0.0167 for paradigms 1–3, according to Bonferroni correction.*

In paradigm 1, where the velocity difference created the duration difference, the patient group showed a significantly different intercept from the control (0.85 vs. –0.15, *p* = 0.008), while the β value did not (*p* = 0.07). Hence, the patient group had a higher probability of selecting “contralesional rotation was longer” than the control group when equal velocity was applied in both directions (0.70 vs. 0.44). In paradigm 2, where a longer duration was assigned to rotation with a small amplitude, the patient group showed an intercept similar to the value of the control group (*p* = 0.68), while the β value significantly differed (*p* = 0.008). The regression slope was inverse of the control group (−0.09 vs. 0.23), indicating that patients with subacute and chronic unilateral vestibulopathy had a loss of precision in estimating duration differences. In paradigm 3, where a longer duration was assigned to rotation with a large amplitude, the intercept and β values were similar between the patient and control groups. In addition, the tendency to increase precision compared to paradigm 1 was also similar between groups.

Statistical model 2, which included velocity differences (ranged from −25°/s to + 25°/s) as a covariate, showed similar results to Model 1, except for paradigm 1, where velocity difference was found to dominate the duration perception (Table 2).

## Discussion

This study evaluated position, velocity/acceleration, and duration perception during whole-body rotation in patients with subacute and chronic unilateral vestibulopathy. There were two main observations.

The first observation was that the patient group showed normal position and velocity/acceleration perception. A previous experiment with repetitive rotations from 90° to 360° with a 90° interval showed that patients with acute and chronic unilateral vestibulopathy had intact position estimates in ipsilesional and contralesional rotations (Cohen et al., 2017). Therefore, the position estimation in this study using more fractionalized intervals reaffirmed the previous findings. Regarding velocity/acceleration perception, the threshold testing with a stepwise rotational velocity increasing paradigm revealed that patients with acute unilateral vestibulopathy recovered the sensitivity only when the vestibular loss was mild (Cousins et al., 2013). However, both in mild and severe cases of vestibular loss, the asymmetry of velocity perception disappeared at the chronic stage (Cousins et al., 2013). The finding was also consistent with the results in this study’s discriminative velocity/acceleration task.

Of interest, the VOR pathway remained compromised in previous and current studies (Cousins et al., 2013; Cohen et al., 2017), which could support that the recovery of the VP pathway is more robust than that of the VOR pathway. The VP and VOR pathways have been known to share a velocity storage circuit (Bertolini et al., 2012). The similarity between VP and VOR imprecision, known to be proportional to stimulus intensity, is another piece of evidence to support a common neural pathway (Nouri and Karmali, 2018). Therefore, the dissociation between the VP and VOR pathways suggests that the perceptual pathway may have additional compensatory neural connections above the brainstem level. In fact, the parietoinsular cortex, the area for vestibular motion perception (Ventre-Dominey, 2014), forms diverse reciprocal connections with cortical and subcortical structures and the contralateral cortex (Brandt et al., 2012; Kirsch et al., 2016), which could play a compensatory role (Dieterich and Brandt, 2015).

The second finding was impaired duration discrimination in the patient group. A previous experiment quantitatively evaluating the duration perception reported a robust resilience of duration perception in patients with unilateral vestibulopathy. Unlike the VOR, the duration perception maintained the symmetricity of duration perception in acute and chronic stages (Cousins et al., 2013). However, our result in paradigm 1 of the duration task in which the velocity difference created the duration difference was different. The probability of selecting “contralesional rotation was faster” was about 0.7 when the duration difference did not exist, suggesting significant inaccuracy in duration perception. In the complex task, where the longer duration rotation was assigned to a larger rotation amplitude (paradigm 3), the duration perception became more precise, as in the control group. However, in the task where the longer duration was assigned to a smaller rotation amplitude (paradigm 2), the precision was significantly lower and worsened than in the control group. Hence, our findings suggest that patients with subacute to chronic unilateral vestibulopathy have an impaired perception of duration in terms of accuracy and precision.

Of interest, there was a noticeable effect of velocity difference or position amplitude in the duration task. The velocity difference was the main factor dominating the duration perception of paradigm 1. The impairment and improvement of duration discrimination in paradigms 2 and 3 would also reflect the effect of position amplitude. These results imply a mathematical equation-like relationship between perceptions of position, velocity, and duration and suggest a duration task’s usefulness in assessing all elements of vestibular perception.

There may be an argument that the findings discussed thus far conflict. Since the temporoparietal cortex has been suggested to compute position from the velocity and duration signals (Kaski et al., 2016), the position estimate cannot be accurate in the impaired duration perception. However, in this study, position perception was evaluated semi-quantitatively, while duration and velocity/acceleration perceptions were evaluated by forced binary choice. Therefore, the errors in the duration perception observed in this study may have been insufficient to cause errors in the positional estimation. In fact, the contralesional duration bias with intact velocity/acceleration perception in this study can explain the contralesional positional bias reported in more complex tasks, such as the repetitive asymmetric rotation paradigm (Panichi et al., 2017).

Clinically, patients with chronic unilateral vestibulopathy often have reported isolated dizziness and spatial misperception without a false motion sense (Bisdorff et al., 2009). Because duration errors without velocity/acceleration misperception can lead to spatial misperception, the study findings may be partly meaningful in interpreting the dizzy symptoms. Although we did not evaluate the velocity and duration perceptions in the same way, there may be a way to determine the error in velocity perception. In that case, we may explain patients with a false motion sense without other signs. Additionally, in this context, different experimental paradigms may also be required to evaluate the VP pathway, like complex VOR testing (e.g., head shaking and skull vibration maneuvers), adopted to reveal the hidden imbalances (Koo et al., 2011; Choi et al., 2016).

Finally, in the previous study on normal subjects, the duration perception was changeable, especially in the elderly, according to the interaction between the amplitude and duration of motion (Choi et al., 2021). This finding may favor the existence of an intrinsic timing mechanism distributed in the vestibular system (Burr et al., 2007) and explain the higher prevalence of dizziness (spatial disorientation without a sense of false motion) in the elderly (Furman et al., 2010). Furthermore, a recent study with the neuropsychological vertigo inventory showed that patients with vestibular disorders had impaired time perception (Xie et al., 2021). Therefore, the altered accuracy (contralesional bias) and precision in duration perception among patients with unilateral vestibulopathy may further support the intrinsic and distributed timing mechanism of the vestibular system.

Our study had several limitations. First, the suprathreshold acceleration could affect vestibular perception. Though the acceleration period adopted in our experiments was set at 0.15 s, the short duration of rotation (e.g., 1 s rotation) would not be completely free from the effect. Second, this study had a small sample size, so further studies must verify our result. Third, we analyzed the regression fit based on the group data due to limited rotation trials per given stimuli. Though we fitted the regression after averaging the response (probability) at each given stimulus first to minimize the large and abnormal effects of a few subjects with poor performance, the results are limited for application to individual patients with vestibular pathology. Further study will be needed to identify and characterize the impaired vestibular perception in vestibulopathy at the individual level. Fourth, the finding could not reflect the specific disease condition because we included patients with variable disease duration and etiology. We can also test our paradigm in the acute and recovery stages of vestibulopathy in future studies. Finally, in the position task, we guided participants to report position estimates at 30° intervals. This prior information may have affected the position estimates and masked small biases in patients. In addition, the simplicity of rotational stimuli could not reveal a small bias. Likewise, the simple velocity/acceleration task protocol might have been insufficient to discover the hidden bias of velocity perception. The rotation signal decays over time despite the velocity-storage compensation. In the velocity/acceleration task, the vestibular signal from low-velocity rotations (10°/s for 30° and 60°) attenuated more than for high-velocity rotations, so participants would have been able to discern the velocity difference more easily. Therefore, developing and applying a more complex protocol in the position and velocity/acceleration tasks is required.

## Conclusion

Vestibular perception may be persistently impaired in the duration domain in patients with unilateral vestibular hypofunction, even when the other domains, such as position and velocity/acceleration perception, remain intact. Complex perceptual tasks may disclose the hidden errors of vestibular perception and partly account for persistent perceptual disturbances in patients with unilateral vestibular hypofunction.

## Data Availability Statement

The original contributions presented in the study are included in the article/supplementary material, further inquiries can be directed to the corresponding author/s.

## Ethics Statement

The studies involving human participants were reviewed and approved by the Institutional Review Board of Seoul National University Bundang Hospital (B-1908-556-301). The patients/participants provided their written informed consent to participate in this study.

## Author Contributions

EK analyzed, interpreted the data, and wrote the manuscript. J-YL, J-MS, H-JK, and J-HL performed the experiments and collected the data. J-YC and J-SK designed, conceptualized the study, interpreted the data, and revised the manuscript. All authors contributed to the article and approved the submitted version.

## Conflict of Interest

The authors declare that the research was conducted in the absence of any commercial or financial relationships that could be construed as a potential conflict of interest.

## Publisher’s Note

All claims expressed in this article are solely those of the authors and do not necessarily represent those of their affiliated organizations, or those of the publisher, the editors and the reviewers. Any product that may be evaluated in this article, or claim that may be made by its manufacturer, is not guaranteed or endorsed by the publisher.
